# Essential fatty acids disrupt the mycolic acid-rich cell envelope of clinical *Nocardia* isolates: a membrane-targeting approach to combat persistent infections

**DOI:** 10.3389/fcimb.2026.1878912

**Published:** 2026-08-07

**Authors:** Ming Wei, Shiwang Xie, Peng Wang, Tianmeng Li, Jun Liu, Jingxian Yang, Xinmin Xu, Dan Luo, Li Gu, Shuai Wang

**Affiliations:** 1 Department of Infectious Diseases and Clinical Microbiology, Beijing Institute of Respiratory Medicine and Beijing Chao-Yang Hospital, Capital Medical University, Beijing, China; 2 CAS Center for Excellence in Nanoscience, Beijing Key Laboratory of Micro-Nano Energy and Sensor, Beijing Institute of Nanoenergy and Nanosystems, Chinese Academy of Sciences, Beijing, China; 3 Department of Clinical Laboratory, Aerospace Center Hospital, Beijing, China; 4 Department of Clinical Laboratory, Beijing Ditan Hospital, Capital Medical University, Beijing, China; 5 National Institutes for Food and Drug Control, State Key Laboratory of Drug Regulatory Science, NHC Key Laboratory of Research on Quality and Standardization of Biotech Products, NMPA Key Laboratory for Quality Research and Evaluation of Biological Products, Beijing, China

**Keywords:** amikacin combination, essential fatty acids, membrane disruption, mycolic acid, *Nocardia*

## Abstract

**Background:**

Conventional antibiotics against slow-growing, mycolic acid-rich *Nocardia* often require prolonged therapy and are associated with high relapse rates. Membrane-targeting essential fatty acids may circumvent growth-dependent resistance.

**Methods:**

Five essential fatty acids were screened against 82 clinical *Nocardia* isolates by broth microdilution. Docosahexaenoic acid (DHA) was further assessed for bactericidal kinetics, membrane integrity by propidium iodide staining and scanning electron microscopy, transcriptomic profiling, and combined activity with amikacin and tobramycin.

**Results:**

α-Linolenic acid, γ-linolenic acid, eicosapentaenoic acid and DHA exhibited comparable activity (MIC_50/_MIC_90_: 31.25/62.50 μM), corresponding to mass concentration ranges of 8.7 to 10.3 μg/mL for MIC_50_ and 17.4 to 20.6 μg/mL for MIC_90_. However, arachidonic acid was markedly less active. DHA rapidly disrupted the *Nocardia* cell envelope, causing propidium iodide influx and ultrastructural damage, and achieved concentration-dependent bactericidal killing within 1 h, a feat not observed with imipenem. Transcriptomic analysis of the surviving subpopulation revealed downregulation of the proton-pumping Nuo complex, upregulation of type II NADH dehydrogenase (Ndh), and global induction of translation machinery. These transcriptional changes, together with pre-existing membrane damage, additively enhanced the activity of amikacin against intrinsically resistant *N. wallacei* (FICI, 0.51-0.56).

**Conclusions:**

These findings demonstrate that DHA displays rapid membrane-disruptive activity and can enhance amikacin activity against *Nocardia* under the defined *in vitro* conditions.

## 1 Introduction

The rise of antimicrobial resistance has exposed a critical limitation of conventional antibiotics: their reliance on active bacterial metabolism (Ahmad et al., 2025). Drugs that inhibit cell wall synthesis, protein translation, or DNA replication are often ineffective against slow-growing or non-replicating bacteria, which persist in chronic infections and evade immune clearance (Fisher et al., 2017; Roemhild et al., 2022). This problem is particularly acute in diseases caused by organisms with inherently long generation times, such as *Nocardia* and *Mycobacterium* species (Daley et al., 2020; Saukkonen et al., 2025; Yetmar et al., 2025). These pathogens frequently establish persistent infections that respond poorly to standard therapy, leading to prolonged treatment courses, high relapse rates, and significant morbidity.

An alternative approach is to target the bacterial cell membrane, a structure essential for viability irrespective of growth state. Agents that physically disrupt membrane integrity can cause rapid depolarization, ion leakage, and cell death without requiring metabolic activity (Hurdle et al., 2011; Ganesan et al., 2023). This mechanism offers a distinct advantage against dormant subpopulations and biofilm-associated bacteria, which are largely refractory to traditional antibiotics. As highlighted in a seminal review by Hurdle and colleagues, membrane-targeting strategies remain underutilized despite their potential to overcome the limitations of current antimicrobial classes (Hurdle et al., 2011).

*Nocardia* species possess a unique cell envelope characterized by a dense layer of mycolic acids, which are long-chain, branched fatty acids that confer extreme hydrophobicity and low permeability (Marrakchi et al., 2014). This lipid-rich barrier protects the bacterium from many hydrophilic antibiotics and host defenses. However, it may also create a vulnerability to lipophilic compounds capable of integrating into and destabilizing the membrane bilayer. Essential fatty acids (EFAs), such as docosahexaenoic acid (DHA), are naturally occurring polyunsaturated lipids that cannot be synthesized *de novo* by humans and must be obtained through diet (Frydrych et al., 2025). Their amphipathic structure enables insertion into lipid membranes, where they perturb fluidity, increase permeability, and compromise barrier function. Importantly, because their action stems from physicochemical disruption rather than binding to a specific molecular target, resistance development is expected to be less frequent (Yoon et al., 2018).

Currently available membrane-active antibiotics have significant limitations. Daptomycin, although effective against many Gram-positive pathogens, demonstrates little to no activity against *Nocardia* species in standard susceptibility testing (Huang et al., 2007). Polymyxins, such as polymyxin B, target the outer membrane of Gram-negative pathogens; however, recent evidence indicates their efficacy depends partially on bacterial respiration and membrane potential, limiting activity against metabolically quiescent cells (Borrelli et al., 2025). In addition, polymyxins are associated with substantial nephrotoxicity and are increasingly compromised by transferable resistance mechanisms (Poirel et al., 2017). In contrast, EFAs have been widely consumed in human diets and clinical supplements for decades, with a strong record of safety and tolerability at physiological and supraphysiological doses.

Nocardiosis, caused by *Nocardia* species, poses a serious threat to immunocompromised patients and often manifests as severe pulmonary or disseminated disease with central nervous system involvement (Du et al., 2025). First-line therapy relies on trimethoprim-sulfamethoxazole, a combination that inhibits two sequential steps in bacterial folate metabolism. This dual blockade is effective primarily against replicating bacteria, and its metabolic dependency contributes to prolonged treatment durations (often 6 to 12 months), suboptimal efficacy against dormant subpopulations, and frequent relapses (Yetmar et al., 2025). Moreover, trimethoprim-sulfamethoxazole is associated with significant adverse effects, including hyperkalemia, nephrotoxicity, hypersensitivity syndrome, and decreased oxygen-carrying capacity (Ho and Juurlink, 2011). Acquired resistance to trimethoprim-sulfamethoxazole is also well documented among clinical Nocardia isolates, further undermining long-term therapeutic success (Mehta et al., 2018; Saksena et al., 2020; Barry et al., 2022; Hershko et al., 2023).

In this study, we evaluated the *in vitro* antimicrobial activity of DHA and other essential fatty acids against a collection of clinical *Nocardia* isolates. We test the hypothesis that these lipids exert concentration-dependent bactericidal effects primarily through disruption of the cell membrane. Our results provide a foundation for exploring EFAs or structurally optimized derivatives as novel agents against infections caused by slow-growing bacterial pathogens with lipid-rich cell walls, such as those containing mycolic acid.

## 2 Materials and methods

### 2.1 Chemicals

Alpha-linolenic acid (ALA), γ-linolenic acid (GLA), eicosapentaenoic acid (EPA), docosahexaenoic acid (DHA), arachidonic acid (AA), and imipenem (IPM) were purchased from Sigma-Aldrich (Missouri, USA). Dimethyl sulfoxide (DMSO), propidium iodide (PI), amikacin, tobramycin and glutaraldehyde were purchased from Solarbio (Beijing, China). Mueller Hinton II broth was obtained from Becton, Dickinson and Company (New Jersey, USA). Columbia blood agar plates were purchased from Thermo Fisher Scientific (Beijing, China).

### 2.2 Bacterial isolates and culture conditions

This study utilized a previously established collection of 82 clinical *Nocardia* isolates. These isolates were recovered between January 2010 and December 2020 from three tertiary hospitals in Beijing: Beijing Chao-Yang Hospital, Beijing Ditan Hospital, and Beijing Aerospace Center Hospital. All isolates had been previously identified to the species level using a multilocus sequence analysis (MLSA) scheme targeting *gyrB*, 16S rRNA, and *secA1* (Wei et al., 2021). The collection comprised 12 distinct species, with *Nocardia cyriacigeorgica* (n = 33, 40.2%) and *N. farcinica* (n = 20, 24.4%) being the most prevalent. Additional species included *N. otitidiscaviarum* (n = 7), *N. abscessus* (n = 5), *N. asiatica* (n = 4), *N. wallacei* (n = 4), *N. puris* (n = 2), and single isolates each of *N. aobensis* (n = 1), *N. brasiliensis* (n = 1), and *N. nova* (n = 1). Notably, three isolates initially designated as novel species I which have since been formally described as *Nocardia sputi* (n = 3) in a separate taxonomic study (Wang et al., 2022). The collection also includes one isolate representing a distinct, putative novel species, designated novel species II (n = 1) in prior MLSA characterization. For the present study, each isolate was recovered from -20 °C storage and passaged twice on Columbia blood agar at 35 °C for 72 hours prior to experimental use.

### 2.3 Evaluation of the antimicrobial activity of EFAs against *Nocardia* isolates

The antimicrobial activity of EFAs (ALA, GLA, EPA, DHA, and AA) was evaluated by broth microdilution. The assay was performed following CLSI guidelines for *Nocardia*, under conditions comparable to those used for standard antibiotic susceptibility testing (CLSI, 2011). Stock solutions of each fatty acid were prepared in DMSO at 2000 mM. These stock solutions were diluted in Mueller-Hinton II broth with vortex mixing for 1 min to achieve a starting concentration of 2000 μM. Serial twofold dilutions were then prepared in Mueller-Hinton II broth to obtain the concentrations ranging from 15.63 to 2000 μM.

*Nocardia* isolates were adjusted to approximately 0.5 McFarland standard and diluted 100-fold in Mueller-Hinton II broth. Fifty microliters of this diluted inoculum were added to each well of a 96-well round-bottom microtiter plate, followed by 50 μL of the serially diluted fatty acid solutions. The final well volume was 100 μL. After mixing, the final concentrations of the essential fatty acids in the assay wells ranged from 7.81 to 1000 μM, and the final DMSO concentration in all MIC wells was below 0.05% (v/v). A DMSO-only control (0.05%, v/v) was included. A growth control and a sterility control were also included. Plates were incubated at 35 °C for 72 h (or up to 120 h if growth in the growth control wells was insufficient after 3 days).

Before reading MICs, all plates were visually inspected for fatty acid precipitation or compound-related turbidity. At a final concentration of 1000 μM, a uniform haze (not due to bacterial growth) was observed; at lower concentrations, no haze or precipitation was seen. The minimum inhibitory concentration (MIC) was defined as the lowest concentration that completely inhibited visible bacterial growth. For each isolate and each EFA, the MIC assay was performed in triplicate.

### 2.4 Evaluation of combined effects of DHA with aminoglycosides against *Nocardia wallacei* isolates

To investigate whether DHA, a representative essential fatty acid, enhances the permeability of aminoglycosides by disrupting the mycolic acid-rich cell envelope, we evaluated the combined antimicrobial activity of DHA with amikacin and tobramycin against *Nocardia wallacei* isolates. *Nocardia wallacei* was specifically selected for combination testing due to its intrinsic high-level resistance to aminoglycosides, a property mediated by the chromosomally encoded 16S rRNA methyltransferase WarA (Lowman and Aithma, 2010; Hershko et al., 2024). Checkerboard broth microdilution assays were performed in 96-well microtiter plates according to established protocols (Li et al., 2025). Briefly, DHA was tested at final concentrations corresponding to 1/4× MIC, 1/2× MIC and 1× MIC for each isolate, while amikacin and tobramycin were tested at final concentrations ranging from 1 to 64 μg/mL and 1 to 16 μg/mL, respectively. Stock solutions were prepared in cation-adjusted Mueller-Hinton II broth at 4× the desired final concentration. A volume of 25 μL of each antimicrobial agent was added to the wells. Bacterial suspensions were adjusted to a 0.5 McFarland standard, diluted 100-fold in broth, and 50 μL was inoculated into each well, resulting in a final volume of 100 μL per well. Plates were incubated at 35 °C for 120 h. The interaction between DHA and each aminoglycoside was assessed using the fractional inhibitory concentration index (FICI), calculated as follows: FICI = (MIC of DHA in combination/MIC of DHA alone) + (MIC of aminoglycoside in combination/MIC of aminoglycoside alone). Synergy was defined as FICI ≤ 0.5, additivity as 0.5 < FICI ≤ 1.0, indifference as 1.0 < FICI ≤ 4.0, and antagonism as FICI > 4.0 (Noel et al., 2021). All assays were performed in triplicate.

### 2.5 Comparative bactericidal activity of DHA and IPM against *N. cyriacigeorgica* CY040

To directly compare the bactericidal effects of DHA and IPM, *N. cyriacigeorgica* CY040 was first cultured on Columbia blood agar at 35 °C for 72 h. Fresh colonies were harvested and suspended in 0.45% sterile saline to a density of 3 McFarland units. The suspension was aliquoted into twelve sterile tubes (1 mL per tube), constituting three biological replicates for each of four treatment groups: DHA (final concentration: 2000.0 μM, equivalent to 657.0 µg/mL), IPM (final concentration: 128.0 µg/mL), a solvent control (0.3 µL DMSO + 0.7 µL saline), and a viability control (1 µL saline only). All tubes were incubated at 35 °C with shaking at 150 rpm for 1 h. After incubation, suspensions from the IPM, solvent control, and viability control groups were serially diluted 10^5^-fold in saline for plating, while the DHA-treated suspension was plated without dilution due to substantial bactericidal activity. From each dilution (or the undiluted DHA sample), 10 µL was spread onto Columbia blood agar plates. Colony-forming units (CFUs) were enumerated after incubation at 35 °C for 72 h.

### 2.6 Concentration-dependent bactericidal activity of DHA against *N. cyriacigeorgica* CY040

The bactericidal activity of DHA was further evaluated across a concentration gradient. A bacterial suspension of *N. cyriacigeorgica* CY040 was prepared in 0.45% sterile saline and adjusted to 3 McFarland units. Aliquots (1 mL) were treated with DHA to achieve final concentrations of 0 (viability control), 125.0, 500.0, and 2000.0 μM, with three biological replicates per concentration. All tubes were then incubated under identical conditions (35 °C, 150 rpm) for 1 h. Following incubation, cultures were subjected to appropriate serial dilutions in saline to yield countable colonies. Subsequently, 10 µL of each dilution was spread onto Columbia blood agar plates. CFUs were counted after a 72 h incubation at 35 °C.

### 2.7 Assessment of cell membrane integrity by confocal microscopy

Bacterial membrane integrity following treatment was evaluated using the membrane-impermeant nucleic acid stain PI. *N. cyriacigeorgica* CY040 suspensions were treated as described in Section 2.5, comprising four groups: DHA (125.0 μM), imipenem (IPM, 128.0 µg/mL), a solvent control (0.3 µL DMSO + 0.7 µL saline), and a viability control (1 µL saline only), followed by incubation at 35 °C with shaking for 1 h. The cells were then pelleted by centrifugation at 5,000 × g for 5 min, washed once, and resuspended in 95 µL of sterile saline. A 5 µL aliquot of a PI solution (100 µg/mL in PBS) was added to each suspension. After incubation in the dark at room temperature for 2 min, 10 µL of the stained suspension was placed on a glass slide for imaging. Observations were performed using a Zeiss LSM 900 confocal laser scanning microscope (Carl Zeiss Meditec AG, Jena, Germany). PI fluorescence was detected using excitation and emission wavelengths of 594 nm and 617 nm, respectively. Corresponding bright-field images were acquired simultaneously and merged with fluorescence images for analysis.

### 2.8 Examination of bacterial ultrastructure by scanning electron microscopy

Morphological alterations in bacterial cells induced by DHA and IPM were examined using scanning electron microscopy (SEM). Bacterial suspensions of *N. cyriacigeorgica* CY040 were prepared and treated under identical conditions as for the confocal microscopy assay (Section 2.7). Post-treatment, cells were harvested by centrifugation (5,000 × g, 5 min). The pellets were fixed with 2.5% glutaraldehyde for 5 h at 4 °C. Following primary fixation, samples were rinsed and subjected to a graded ethanol series (30%, 50%, 70%, 80%, 90%, and 100%) for dehydration. This was followed by two sequential 15-min incubations in fresh 100% ethanol. The dehydrated samples were critically point-dried, sputter-coated with a thin layer of gold, and subsequently imaged using a Hitachi SU8020 SEM (Hitachi High-Technologies Corporation, Tokyo, Japan).

### 2.9 Transcriptomic analysis of the bactericidal mechanism

#### 2.9.1 Isolate selection

*N. cyriacigeorgica* was the most prevalent species in our clinical isolate collection. Therefore, this species was chosen as the representative for transcriptomic analysis. Among the 33 N*. cyriacigeorgica* isolates, the CY040 was selected because its MLSA sequence profile (based on *gyrB*, 16S rRNA, and *secA1*) showed the highest identity to the reference genome *N. cyriacigeorgica* GUH-2 (GenBank accession: GCA_000284035.1), which was the only available reference genome for this species at the time of analysis.

#### 2.9.2 RNA extraction

To elucidate the molecular mechanisms underlying the bactericidal activity of DHA, a comparative transcriptomic analysis was performed. *N. cyriacigeorgica* CY040 suspensions were treated with either DHA at a final concentration of 125.0 μM or an equivalent volume of solvent control (0.3 µL DMSO + 0.7 µL saline), as described in Section 2.6, followed by incubation at 35 °C with shaking for 1 h. Cells were then harvested by centrifugation at 5,000 × g for 5 min at 4 °C. Total RNA was extracted from the bacterial pellets. Briefly, cells were first ground in liquid nitrogen, and RNA was subsequently isolated using an RNA extraction reagent kit (Tiangen Biotech, Beijing, China) according to the manufacturer’s instructions. RNA concentration and purity were assessed using a NanoDrop 2000 spectrophotometer (Thermo Fisher Scientific, Massachusetts, USA), and RNA integrity was verified using a Bioanalyzer 2100 system (Agilent Technologies, California, USA). Only RNA samples with an A260/A280 ratio between 1.8 and 2.0 and an RNA Integrity Number (RIN) greater than 8.0 were used for downstream library construction and sequencing.

#### 2.9.3 RNA sequencing and analysis

RNA sequencing was conducted by Novogene Co., Ltd. (Beijing, China) on an Illumina NovaSeq 6000 platform using a PE150 strategy, generating paired-end reads with an average length of 150 bp.

For transcriptomic analysis, raw paired-end reads were first quality-filtered and adapter-trimmed using Fastp (v0.19.7) with default parameters. The resulting clean reads were then aligned to the reference genome of *Nocardia cyriacigeorgica* GUH-2 using Bowtie2 (version 2.5.4). Gene quantification was performed using featureCounts (v2.0.6) to assign mapped reads to genomic features. The expression level of each gene was then quantified as Fragments Per Kilobase of transcript per Million mapped reads (FPKM), calculated based on the gene length and its corresponding read count. Differential gene expression analysis was performed using the DESeq2 R package (v1.42.0). The Benjamini–Hochberg method was applied to adjust the resulting *P*-values for multiple testing, controlling the false discovery rate (FDR). Significantly differentially expressed genes (DEGs) were defined as those with *P*adj < 0.05 and |log_2_FC| ≥ 1, where FC denotes fold change.

Gene ontology (GO) enrichment analysis and Kyoto Encyclopedia of Genes and Genomes (KEGG) pathway analysis of the DEGs were performed using the ClusterProfiler package (v4.8.1). All analyses were based on data from three independent biological replicates per condition.

## 3 Results

### 3.1 Antimicrobial activity of essential fatty acids against clinical *Nocardia* isolates

The *in vitro* antimicrobial activity of five EFAs, ALA, GLA, EPA, DHA, and AA, was evaluated against 82 clinical *Nocardia* isolates representing 12 distinct species using the broth microdilution method. As summarized in **Table 1**, ALA, GLA, EPA, and DHA each exhibited MIC ranges of 15.63-125.0 μM across the tested panel. The MIC_50_ and MIC_90_ values for all four EFAs were uniformly 31.25 μM and 62.50 μM, respectively. Based on molecular weight calculations, these values correspond to mass concentration ranges of 8.7-10.3 μg/mL (MIC_50_) and 17.4-20.6 μg/mL (MIC_90_). Notably, DHA was the only EFA for which any *N. farcinica* isolates were inhibited at 15.63 μM; the MIC minima for the other four EFAs were uniformly 31.25 μM. Consequently, DHA exhibited a broader MIC range (15.63-62.50 μM) against this species, suggesting enhanced potency against a subset of isolates.

**Table 1:** *In vitro* activity of five essential fatty acids against clinically isolated *Nocardia* species.

Species (no. of isolates tested)	Antimicrobial agents (MIC range or value, μM)				
	ALA	GLA	EPA	DHA	AA
*N. cyriacigeorgica* (33)	31.25-62.50	15.63-62.50	15.63-125.0	15.63-125.0	31.25- > 1000.0
MIC_50/90_ ^a^	62.50/62.50	31.25/62.50	31.25/62.50	62.50/62.50	250.0/500.0
*N. farcinica* (20)	31.25-62.50	31.25-62.50	31.25-62.50	15.63-62.50	31.25-1000.0
MIC_50/90_	31.25/62.50	31.25/62.50	31.25/31.25	31.25/31.25	250.0/500.0
*N. otitidiscaviarum* (7)	31.25-125.0	31.25-125.0	31.25-125.0	31.25-125.0	250.0/1000.0
MIC_50/90_	62.50/125.00	31.25/125.00	31.25/125.0	31.25/125.0	250.0/1000.0
*N. abscessus* (5)	31.25	15.63-31.25	15.63-31.25	15.63	31.25
MIC_50/90_	31.25/31.25	31.25/31.25	15.63/31.25	15.63/15.63	31.25/31.25
*N. asiatica* (4) ^b^	31.25	15.63-31.25	15.63-31.25	15.63-31.25	31.25-125.0
*N. wallacei* (4)	31.25-62.50	15.63-31.25	15.63-31.25	31.25-62.50	31.25-500.0
*N. sputi* (3)	15.63-31.25	15.63-31.25	15.63	15.63	31.25
*N. puris* (2)	62.50	31.25	31.25	31.25	125.0-250.0
*N. aobensis* (1)	62.50	31.25	31.25	62.50	> 1000.0
*N. brasiliensis* (1)	31.25	31.25	31.25	31.25	125.0
*N. nova* (1)	62.50	62.50	62.50	62.50	500.0
Novel species II (1)	31.25	15.63	15.63	15.63	31.25
All *Nocardia* isolates (82)	15.63-125.0	15.63-125.0	15.63-125.0	15.63-125.0	31.25- > 1000.0
MIC_50/90_	31.25/62.50	31.25/62.50	31.25/62.50	31.25/62.50	125.0/500.0

^a^ MIC_50/90_, MIC for 50% and 90% of the isolates, respectively.

^b^ For species with fewer than five isolates, MIC_50_ and MIC_90_ were not calculated.

MIC, minimum inhibitory concentration; ALA, α-linolenic acid; GLA, γ-linolenic acid; EPA, eicosapentaenoic acid; DHA, docosahexaenoic acid; AA, arachidonic acid.

In contrast, AA demonstrated markedly reduced and more variable activity, with MIC values ranging from 31.25 to > 1000.0 μM across the collection. The MIC_50_ and MIC_90_ for AA were 125.0 μM and 500.0 μM, respectively, substantially higher than those observed for the other EFAs. Species-level analysis revealed that *N. abscessus*, *N. sputi*, and the putative novel species II displayed MIC values as low as 15.63 μM for DHA. In contrast, *N. aobensis* and *N. nova* exhibited relatively higher MICs across multiple EFAs, with *N. aobensis* showing MIC value exceeding 1000.0 μM for AA.

### 3.2 Combined activity of DHA with aminoglycosides against *Nocardia wallacei*

The combined activity of DHA with amikacin or tobramycin was evaluated against four *Nocardia wallacei* isolates (CY011, CY028, CY030, and CY063) using a checkerboard broth microdilution method (**Table 2**). For the combination of DHA with amikacin, when tested at 1/4× the MIC of DHA, the MIC of amikacin remained unchanged at 16 μg/mL for isolates CY011 and CY028, decreased from 16 to 8 μg/mL for CY030, and remained at ≥ 128 μg/mL for CY063. At 1/2× the MIC of DHA, the MIC of amikacin was reduced to ≤ 1 μg/mL for all four isolates. The FICI values for the amikacin-DHA combination were 0.56 for CY011, CY028, and CY030, and 0.51 for CY063, all of which fell within the additivity range (0.5 < FICI ≤ 1.0). For the combination of DHA with tobramycin, at 1/4× the MIC of DHA, the MIC of tobramycin remained at ≥ 32 μg/mL for all isolates. At 1/2× the MIC of DHA, the tobramycin MIC remained at ≥ 32 μg/mL for CY011, CY028, and CY030, yielding FICI values of 2, which were interpreted as indifference (1 < FICI ≤ 4). For isolate CY063, however, the tobramycin MIC was reduced from ≥ 32 to ≤ 1 μg/mL at 1/2× the MIC of DHA, resulting in an FICI of 0.51, consistent with additivity.

**Table 2:** *In vitro* combined activity of DHA combined with amikacin and tobramycin against *Nocardia wallacei* isolates.

Parameter	MIC			
	CY011	CY028	CY030	CY063
AK alone (μg/mL)	16	16	16	≥ 128
TOB alone (μg/mL)	≥ 32	≥ 32	≥ 32	≥ 32
DHA alone (μM)	62.50	31.25	31.25	62.50
AK + 1/4× MIC of DHA (μg/mL)	16	16	8	≥ 128
AK + 1/2× MIC of DHA (μg/mL)	≤ 1	≤ 1	≤ 1	≤ 1
FICI (AK + DHA)	0.56	0.56	0.56	0.51
Interpretation (AK + DHA)	additivity	additivity	additivity	additivity
TOB + 1/4× MIC of DHA (μg/mL)	≥ 32	≥ 32	≥ 32	≥ 32
TOB + 1/2× MIC of DHA (μg/mL)	≥ 32	≥ 32	≥ 32	≤ 1
FICI (TOB + DHA)	2	2	2	0.51
Interpretation (TOB + DHA)	indifference	indifference	indifference	additivity

For FICI calculation, MIC endpoints reported as ≤ 1 were defined as 1, while those reported as ≥ 32 and ≥ 128 were defined as 32 and 128, respectively. AK, amikacin; TOB, tobramycin; FICI, fractional inhibitory concentration index.

### 3.3 Comparative bactericidal activity of DHA and imipenem

**Figure 1:**
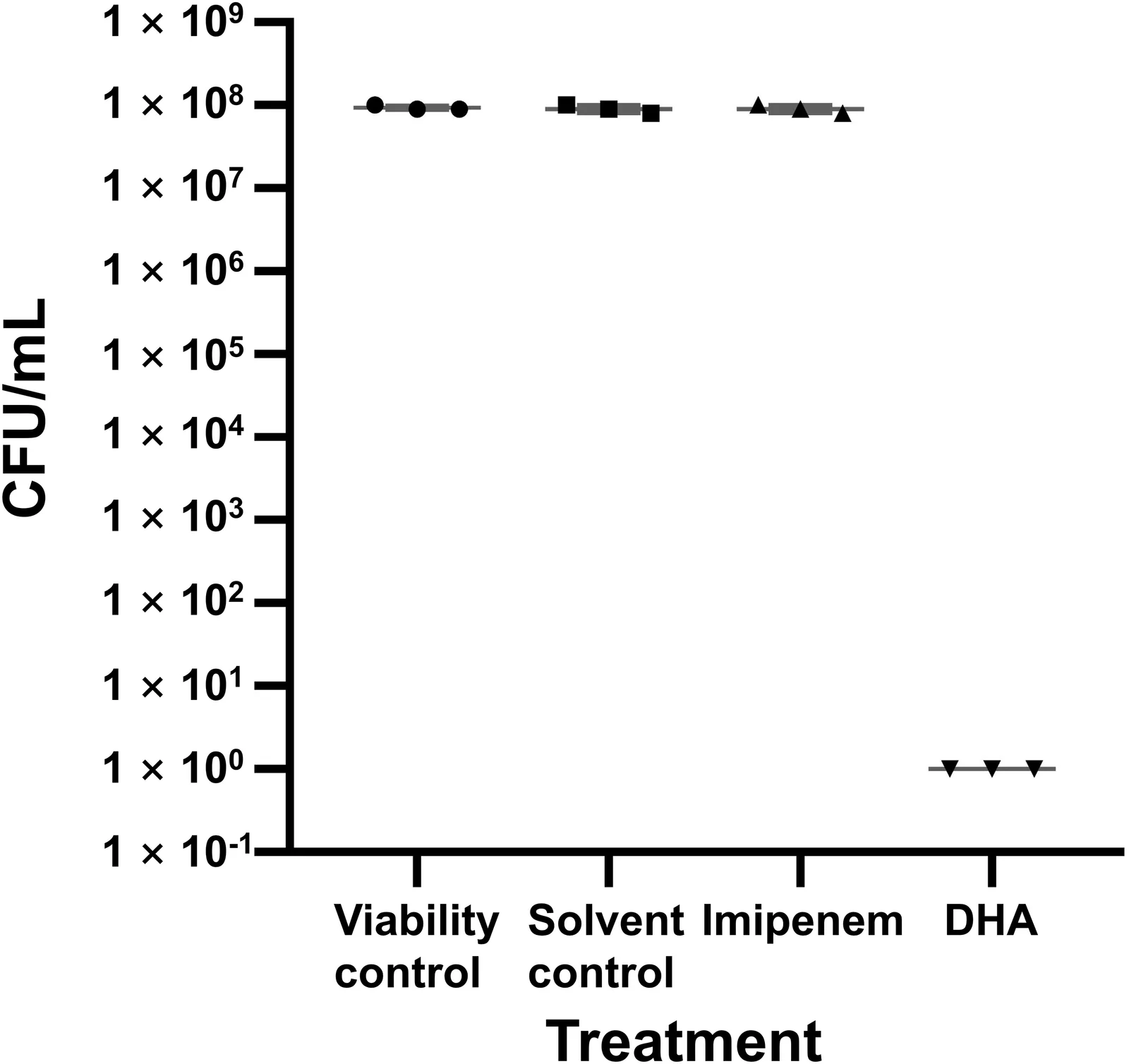
Bactericidal activity of DHA against *N. cyriacigeorgica* CY040 after 1-h treatment. Groups: viability control, solvent control, imipenem, and DHA. For the DHA group, no colonies were detected; these values are plotted at the detection limit (1 CFU/mL) to enable logarithmic display.

To directly compare the bactericidal efficacy of DHA with that of a conventional cell wall-targeting antibiotic, *N. cyriacigeorgica* CY040 was exposed to DHA (2000.0 μM, equivalent to 657.0 µg/mL), imipenem (128.0 μg/mL), solvent control, or viability control for 1 h. Viable counts were enumerated after 72 h of incubation (**Figure 1**).

DHA treatment resulted in complete eradication, with no colonies recovered from any of the three replicates (**Figure 1**). In contrast, the mean viable counts for the viability control, solvent control, and imipenem groups were (9.3 ± 0.57) × 10^7^ CFU/mL, (9.0 ± 1.00) × 10^7^ CFU/mL, and (9.0 ± 1.00) × 10^7^ CFU/mL, respectively. One-way ANOVA revealed no statistically significant difference among these three groups (*P* = 0.870), indicating that imipenem, even at a concentration (128.0 μg/mL) far exceeding its MIC for this isolate (≤ 2 μg/mL), failed to achieve detectable killing within the 1-h exposure period.

### 3.4 Concentration-dependent bactericidal activity of DHA

**Figure 2:**
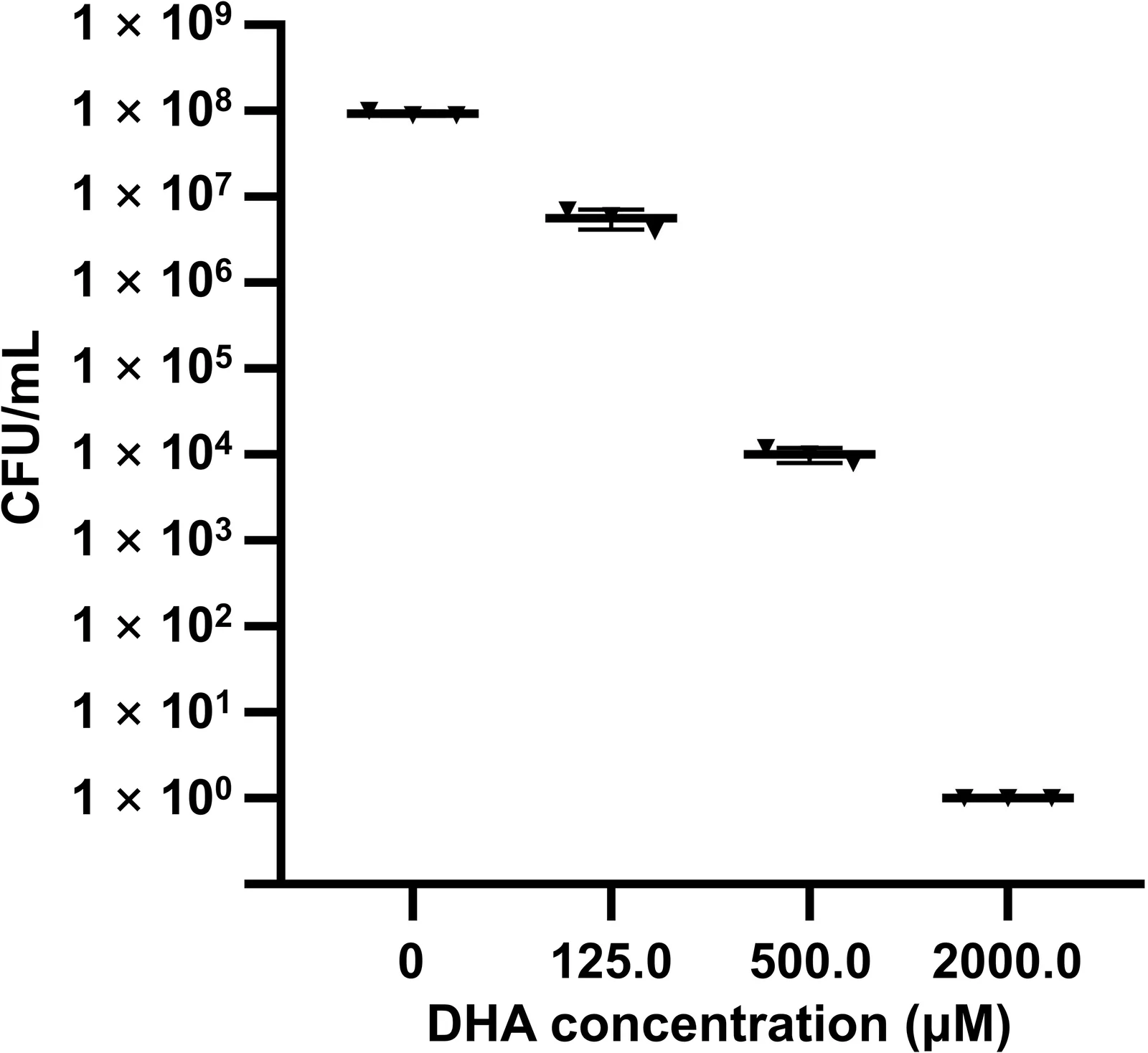
Concentration-dependent bactericidal activity of DHA against *N. cyriacigeorgica* CY040. The Y-axis is shown on a logarithmic scale. For the 2000.0 μM group, no colonies were detected; these values are plotted at the detection limit (1 CFU/mL) to enable logarithmic display.

The bactericidal activity of DHA was further characterized across a concentration gradient. *N. cyriacigeorgica* CY040 was treated with DHA at final concentrations of 125.0, 500.0, and 2000.0 μM for 1 h. Quantitative culture analysis revealed a pronounced concentration-dependent effect: at 125.0 μM, DHA achieved an approximately 1.22-log reduction in viable counts relative to controls; at 500.0 μM, the reduction reached 3.97 logs; and at 2000.0 μM, complete bacterial eradication was observed, with no detectable viable cells (**Figure 2**). These results indicate that DHA exhibits concentration-dependent bactericidal activity against *N. cyriacigeorgica* CY040, with maximal efficacy achieved at 2000.0 μM under the conditions tested.

### 3.5 DHA disrupts bacterial membrane integrity

**Figure 3:**
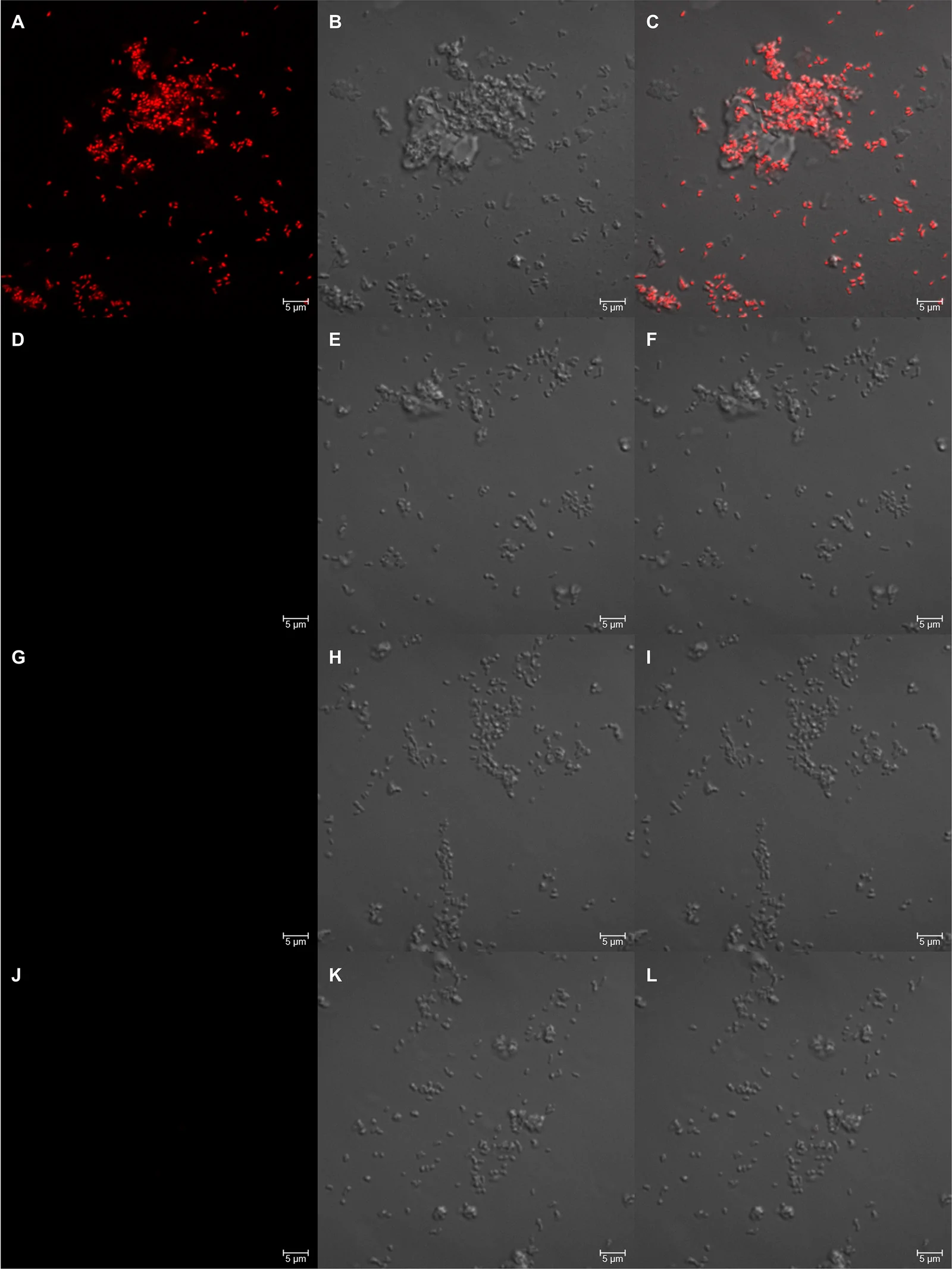
Confocal microscopy images of *N. cyriacigeorgica* CY040 stained with propidium iodide (PI). Cells were treated for 1 h with DHA [125.0 μM; **(A–C)**], imipenem [128.0 μg/mL; **(D–F)**], solvent control [0.3 μL DMSO + 0.7 μL saline; **(G–I)**], or viability control [saline only; **(J-L)**]. For each treatment group, images show PI fluorescence (red; left panels), bright-field (middle panels), and merged fluorescence/bright-field (right panels).

To determine whether the bactericidal activity of DHA was associated with membrane damage, bacterial membrane integrity was assessed using propidium iodide (PI), a membrane-impermeant nucleic acid stain that fluoresces upon entering cells with compromised membranes. Following 1 h of treatment, the majority of DHA-exposed cells exhibited intense red fluorescence, indicating substantial PI uptake and loss of membrane integrity; only a minor subpopulation remained non-fluorescent. (**Figures 3A–C**). In contrast, cells treated with imipenem, solvent control, or viability control showed negligible fluorescence, indicating that intact membrane barriers that prevented PI penetration (**Figures 3D–L**). These observations strongly suggest that DHA exerts its bactericidal effect through direct disruption of the cytoplasmic membrane, whereas imipenem which inhibits cell wall synthesis did not compromise membrane integrity within the same time frame.

### 3.6 DHA induces ultrastructural damage to the cell envelope

**Figure 4:**
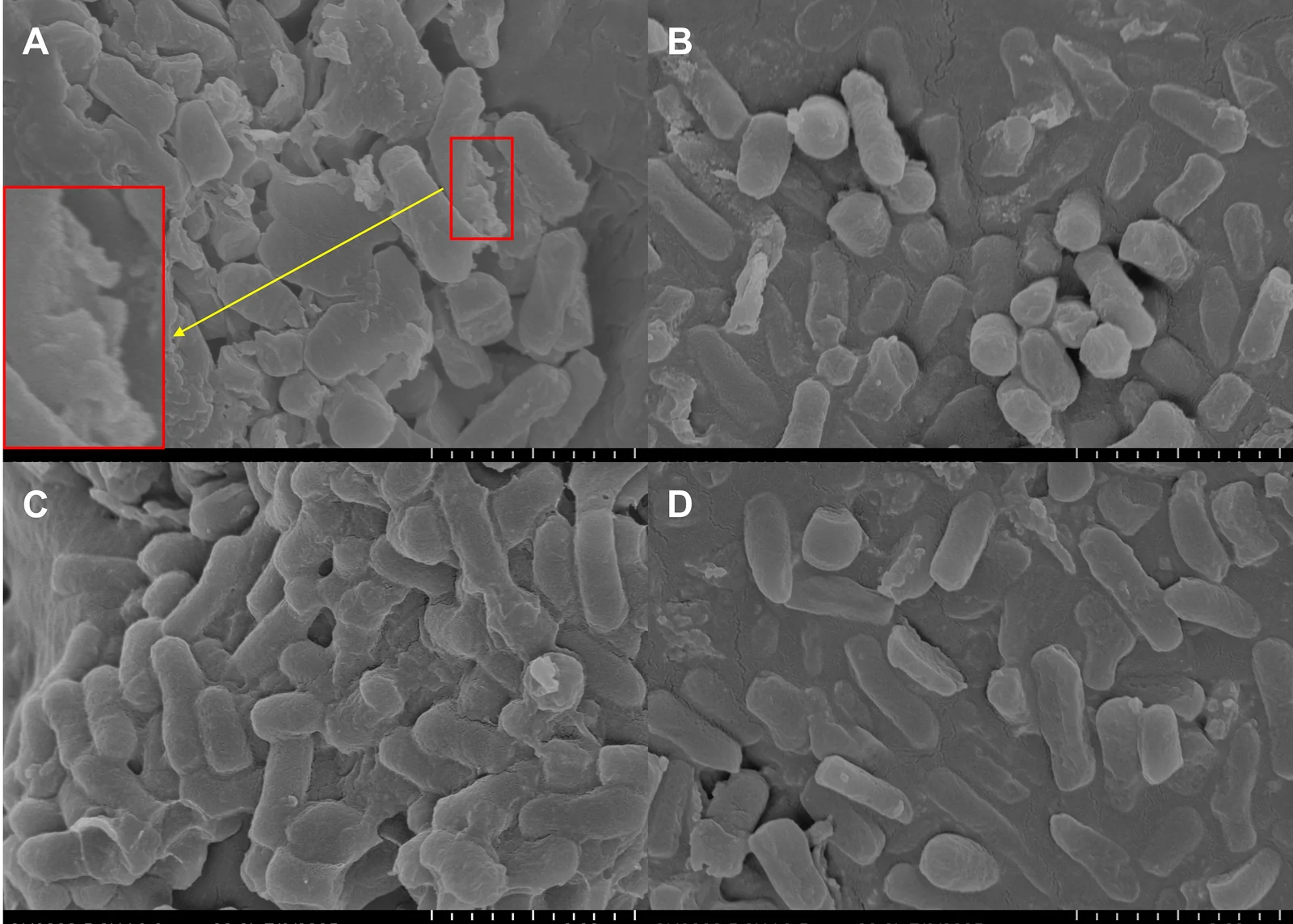
Scanning electron microscopy (SEM) images of *N. cyriacigeorgica* CY040 after 1 h of treatment. **(A)** DHA, with a higher-magnification inset (red square, enlarged view at bottom left), **(B)** imipenem, **(C)** solvent control, and **(D)** viability control groups. Scale bar: 2 μm. Each minor division represents 200 nm.

Morphological alterations induced by DHA and imipenem were visualized using SEM. Viability control cells and those exposed to solvent or imipenem displayed characteristic rod-shaped morphology with smooth, intact cell surfaces and well-defined edges (**Figures 4B–D**). In marked contrast, DHA-treated cells exhibited substantial ultrastructural damage, including irregular surface contours, membrane blebbing, and loss of peripheral structural integrity (**Figure 4A**). The observed frayed-edge morphology is consistent with physicochemical disruption of the cell envelope. These ultrastructural observations provide direct visual evidence of DHA-induced envelope damage, corroborating the membrane-targeting mechanism previously indicated by PI staining.

### 3.7 Transcriptomic analysis

#### 3.7.1 A high-biomass model captures the transcriptional response to DHA

To enable transcriptomic analysis of *Nocardia* under DHA stress, cultures were treated at a density approximately 1000-fold higher than that used in standard MIC assays. The DHA concentration was maintained at 125.0 μM (4× MIC). Under these conditions, approximately 6% of the initial inoculum survived, yielding sufficient RNA for sequencing and capturing both bactericidal effects and transcriptional programs.

#### 3.7.2 Volcano plot analysis of DEGs

The average total mapping rate was 96.01% ± 0.01% for DHA-treated samples and 93.50% ± 0.49% for controls, with uniquely mapped reads averaging 89.51% ± 0.03% and 84.38% ± 5.67%, respectively.

**Figure 5:**
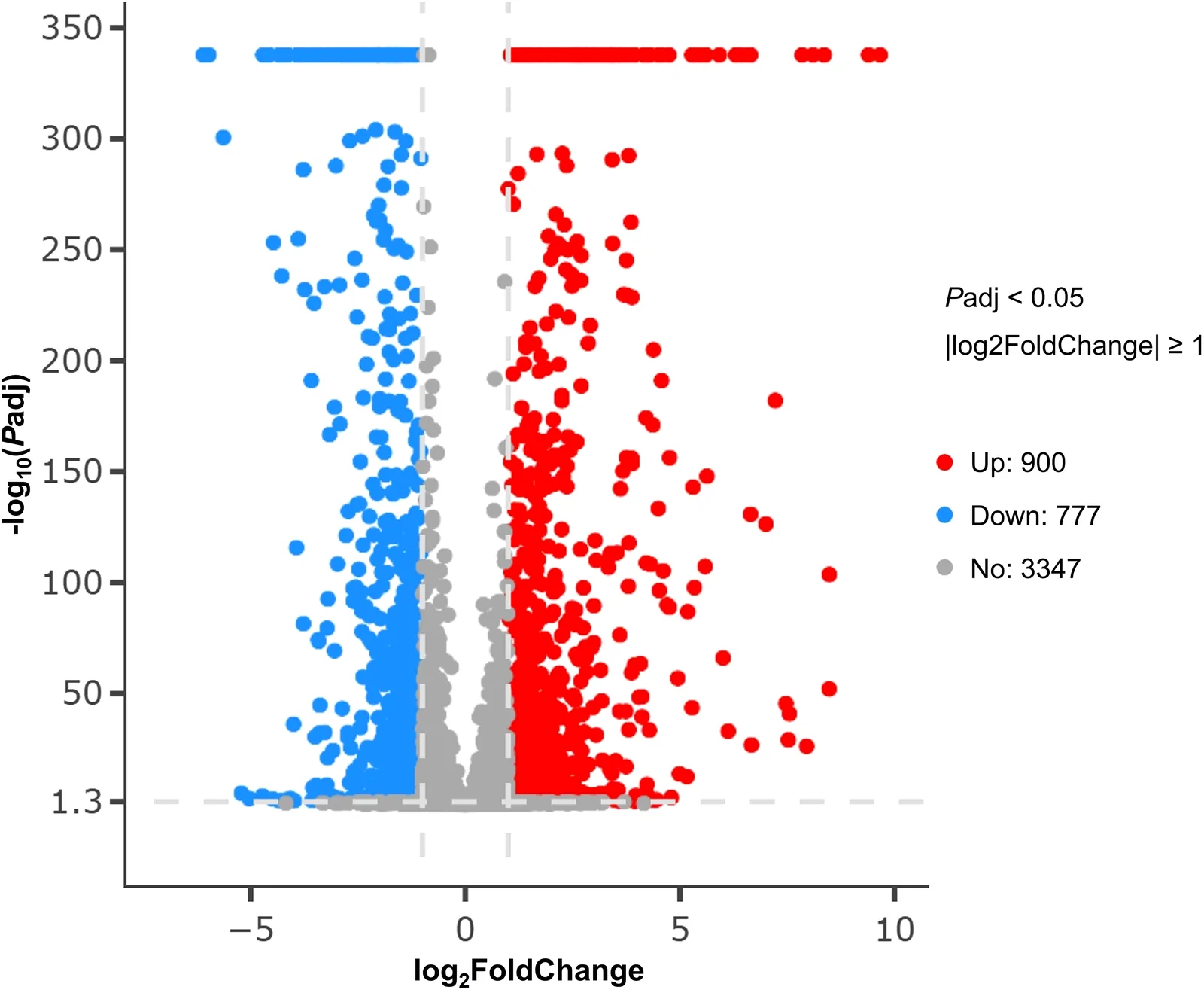
Volcano plot depicting genome-wide differential gene expression in *N. cyriacigeorgica* CY040 following 1 h exposure to DHA compared with solvent control.

The volcano plot (**Figure 5**) provides a comprehensive overview of the global transcriptional response of *Nocardia* to DHA treatment, illustrating the distribution of DEGs across the entire transcriptome. A total of 1,677 genes exhibited statistically significant changes in expression (*P*adj < 0.05 and |log_2_FC| ≥ 1), with 900 genes upregulated (red dots) and 777 genes downregulated (blue dots). The remaining 3,347 genes showed no significant change (gray dots).

#### 3.7.3 DHA induces a global upregulation of biosynthetic genes

**Figure 6:**
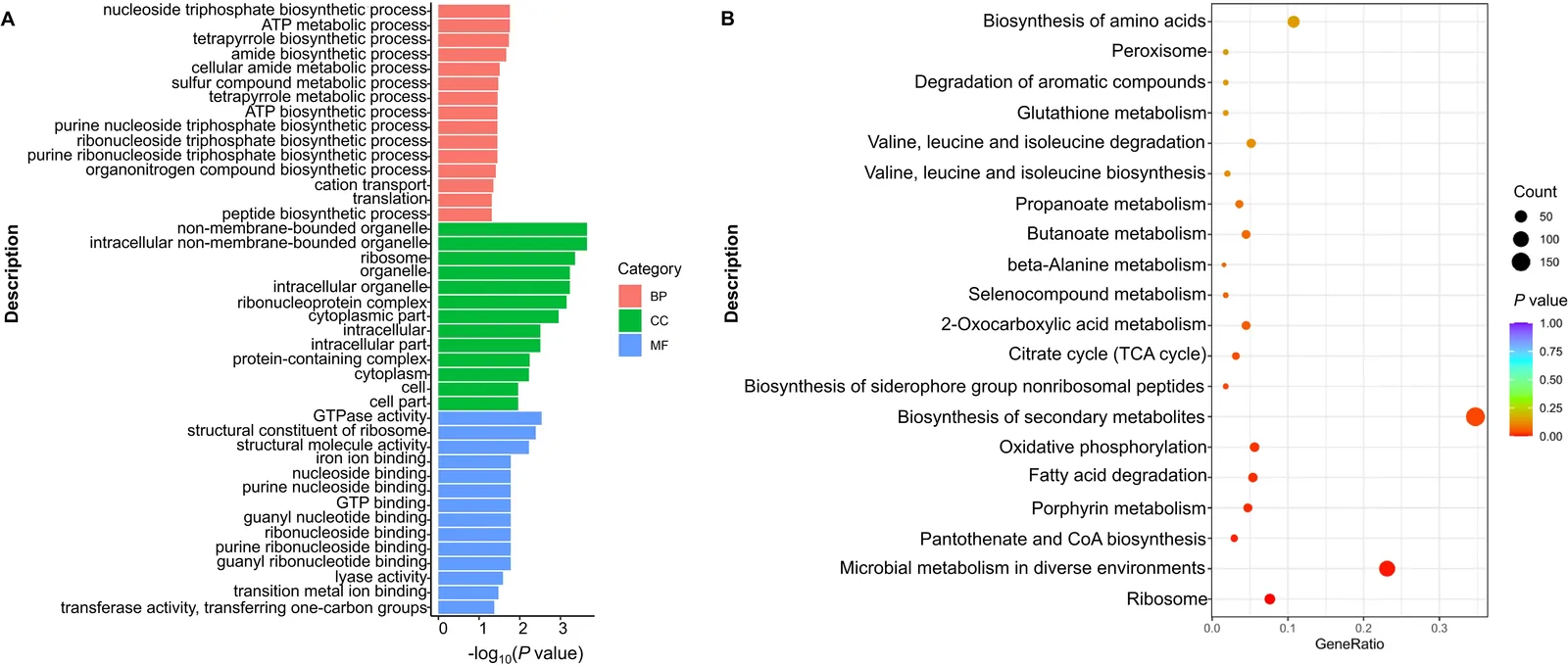
**(A)** Gene ontology (GO) enrichment analysis of differentially expressed genes (DEGs) in DHA-treated *N. cyriacigeorgica* CY040. BP, biological process; CC, cellular component; MF, molecular function. **(B)** Kyoto Encyclopedia of Genes and Genomes (KEGG) pathway enrichment analysis of the same DEGs set.

GO enrichment analysis demonstrated that DHA treatment broadly upregulated anabolic processes (**Figure 6A**; **Supplementary Table 1**). The most significantly enriched biological processes were predominantly associated with protein synthesis and amide metabolism, including amide biosynthetic process (GO:0043604; 37 of 37 DEGs upregulated, 100%), cellular amide metabolic process (GO:0043603; 38 of 38 DEGs upregulated, 100%), translation (GO:0006412; 33 of 33 DEGs upregulated, 100%), and peptide biosynthetic process (GO:0043043; 33 of 33 DEGs upregulated, 100%). In parallel, organonitrogen compound biosynthetic process (GO:1901566; 68 of 75 DEGs upregulated, 90.7%) and sulfur compound metabolic process (GO:0006790; 8 of 10 DEGs upregulated, 80.0%) were also significantly enriched.

Cellular component analysis revealed that the enriched and upregulated terms were predominantly organelle-related. These included organelle (GO:0043226; 27 of 27 DEGs upregulated, 100%), intracellular organelle (GO:0043229; 27 of 27 DEGs upregulated, 100%), non-membrane-bounded organelle (GO:0043228; 27 of 27 DEGs upregulated, 100%), and intracellular non-membrane-bounded organelle (GO:0043232; 27 of 27 DEGs upregulated, 100%). In addition, the upregulation of ribonucleoprotein complex (GO:1990904; 26 of 26 DEGs upregulated, 100%) and its core constituent, the ribosome (GO:0005840; 26 of 26 DEGs upregulated, 100%), paralleled the upregulation of translation-related biological processes. Consistently, KEGG pathway analysis revealed upregulation of the ribosome pathway (ncy03010; 34 of 34 DEGs upregulated, 100%).

At the molecular function level, structural molecule activity (GO:0005198; 27 of 27 DEGs upregulated, 100%) and structural constituent of ribosome (GO:0003735; 27 of 27 DEGs upregulated, 100%) were significantly enriched, consistent with the upregulation of translation-related biological processes and ribosomal components described above. In addition, transferase activity transferring one-carbon groups (GO:0016741; 17 of 23 DEGs upregulated, 73.9%) and GTPase activity (GO:0003924; 6 of 9 DEGs upregulated, 66.7%) were also enriched.

#### 3.7.4 DHA reprograms respiratory gene expression

KEGG pathway analysis of DEGs (**Figure 6B**; **Supplementary Table 2**) revealed significant alterations in oxidative phosphorylation (KEGG ID: ncy00190, 18 of 25 DEGs upregulated, 72.0%). Within this pathway, a distinct functional divergence was observed. Genes encoding the proton-translocating Type I NADH dehydrogenase (Nuo complex) were significantly suppressed (e.g., *nuoA*: log_2_FC = -2.98; FC, fold change; *nuoB*: log_2_FC = -2.75; **Supplementary Table 3**), whereas the non-proton-pumping type II NADH dehydrogenase encoded by *ndh* was significantly upregulated (log_2_FC = 4.51; **Supplementary Table 3**).

#### 3.7.5 DHA alters expression of mycolic acid biosynthesis genes

**Figure 7:**
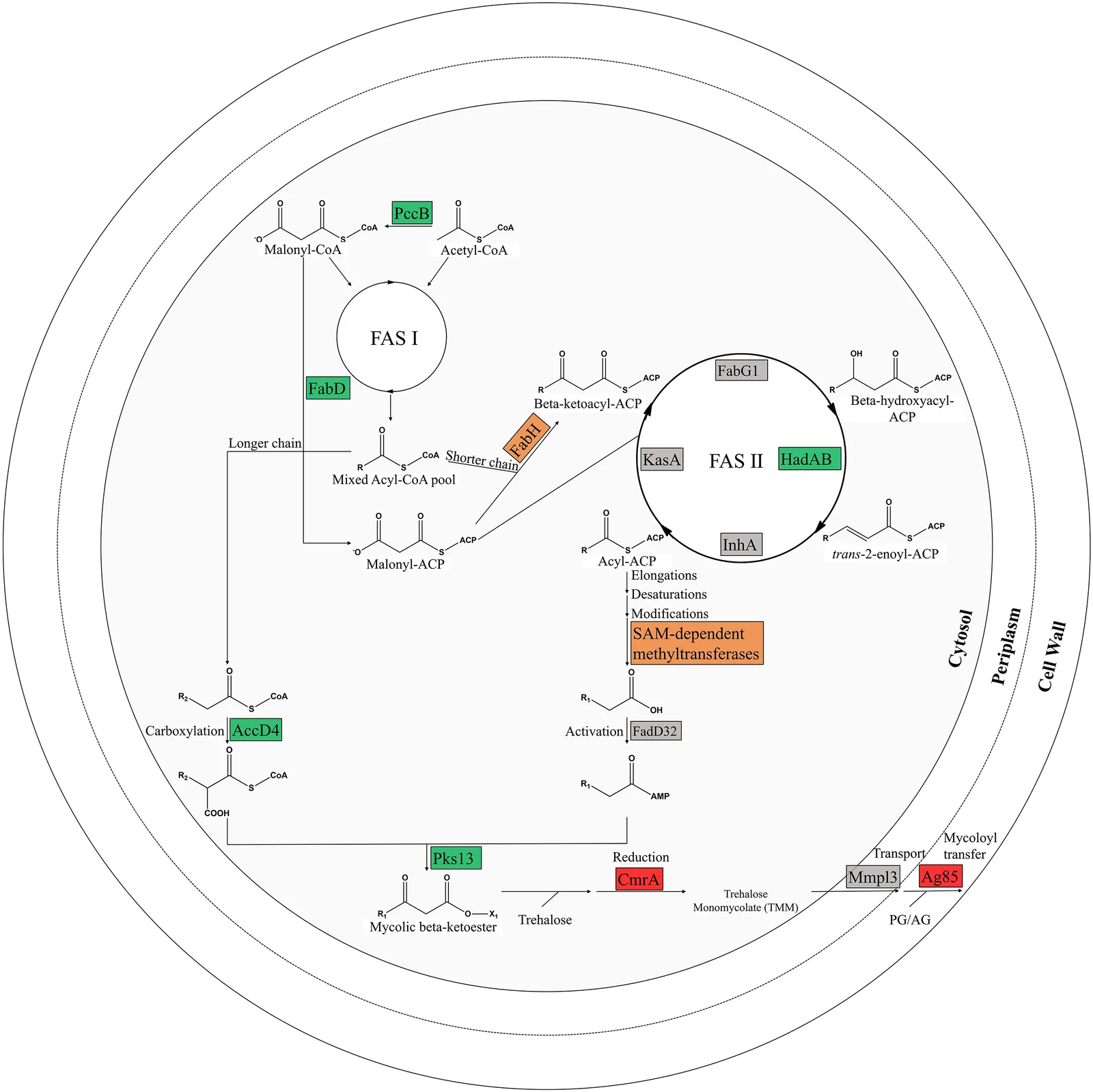
Schematic representation of the mycolic acid biosynthesis pathway in *Nocardia* (adapted from the (Marrakchi et al., 2014), overlaid with transcriptional changes induced by DHA. Expression levels are color-coded according to log_2_FoldChange: red indicates upregulation, blue indicates downregulation, gray indicates no significant change, and orange indicates mixed expression.

DHA treatment significantly altered the expression of genes involved in mycolic acid biosynthesis, suggesting a distinct expression pattern centered around the mycolic β-ketoester formation step (**Figure 7**). Genes functioning upstream of this nodal point were predominantly downregulated (**Supplementary Table 4**). The propionyl-CoA carboxylase subunit gene *pccB* (log_2_FC = -1.27) and the acyl-CoA carboxylase subunit beta gene *accD4* (log_2_FC = -1.71) were downregulated. The FAS-II elongation module was broadly downregulated, including the acyl carrier protein gene *acpM* (log_2_FC = -1.15), the ACP S-malonyltransferase gene *fabD* (log_2_FC = -1.19), two homologous copies of the (3R)-hydroxyacyl-ACP dehydratase genes *hadA/hadB* (log_2_FC = -3.27 and -3.07), and the polyketide synthase gene *pks13* (log_2_FC = -1.19).

Conversely, genes functioning downstream of this biosynthetic node were generally upregulated, including the mycolate reductase gene *cmrA* (log_2_FC = 1.24), and two members of the antigen 85 (*ag85C*) mycolyltransferase gene family (log_2_FC = 2.26 and 2.52). Genes encoding S-adenosylmethionine (SAM)-dependent methyltransferases exhibited mixed expression patterns: several were downregulated, whereas others were upregulated.

## 4 Discussion

This study demonstrates that essential fatty acids, exemplified by DHA, combat clinical *Nocardia* isolates through two functionally distinct consequences of envelope perturbation: rapid bactericidal killing driven by direct physical disruption of the *Nocardia* cell envelope, and a distinct transcriptional response in the surviving subpopulation of the representative isolate *N. cyriacigeorgica* CY040 that occurs independently of primary lethality. By employing a high-biomass culture condition that allowed obtaining sufficient RNA from a surviving subpopulation, we characterized the transcriptional response of these cells.

The mass concentrations corresponding to the MIC_50_ and MIC_90_ of ALA, GLA, EPA, and DHA were 8.7-10.3 μg/mL and 17.4-20.6 μg/mL, respectively. These values are nearly equivalent to the antibacterial activity of conventional β-lactam antibiotics against *Nocardia*, as exemplified by the resistance breakpoints of imipenem (≥ 16 μg/mL), ceftriaxone (≥ 64 μg/mL), and cefepime (≥ 32 μg/mL) (CLSI, 2011). In our previous works, essential fatty acids (EFAs) showed substantially weaker activity against non-mycolate bacteria and fungi, including *Enterococcus faecium*, *Enterococcus faecalis*, and *Candida albicans* (Wang et al., 2022; Wei et al., 2023; Li et al., 2025). Among the tested EFAs, DHA exhibited the strongest activity against *E. faecium*, yet its MIC_50_ and MIC_90_ values were 250 μM and 500 μM, respectively. These values are markedly higher than those observed for *Nocardia* (MIC_50/_MIC_90_: 31.25/62.50 μM), representing an approximately 8-fold difference in susceptibility. This comparison is consistent with the idea that the presence of a mycolic acid-rich envelope may contribute to the sensitivity of *Nocardia* to EFAs.

In our *in vitro* assays, ω-3 fatty acids (ALA, EPA, DHA) exhibited potent killing of clinical *Nocardia* isolates, whereas the ω-6 fatty acid AA showed the weakest activity. This ω-3 versus ω-6 dichotomy is consistent with observations from a murine tuberculosis model, in which an ω-3-enriched diet enhanced pathogen clearance while an ω-6-enriched diet promoted persistence (Jordao et al., 2008). The convergence of these findings across *Nocardia* (direct killing) and *Mycobacterium* (dietary effect) suggests that ω-3 fatty acids act through direct membrane destabilization, as supported by our propidium iodide and electron microscopy data. In contrast, the ω-6 fatty acid AA may be metabolized into mediators that inadvertently support bacterial survival, as inferred from the murine model. Taken together, our results and previous *in vivo* evidence indicate the potential of ω-3 fatty acids as both direct antimicrobials and immunometabolic adjuvants against mycolate-containing pathogens.

The rapid eradication of *N. cyriacigeorgica* by DHA within one-hour contrasts sharply with the inactivity of imipenem under identical conditions despite its low minimum inhibitory concentration. This dichotomy reflects the dependence of conventional β-lactams on active cell wall synthesis during bacterial growth and division, a process that is inherently slow in *Nocardia* (Cho et al., 2014; Traxler et al., 2022). In contrast, DHA integrates immediately into the lipid bilayer as demonstrated by propidium iodide influx and SEM showing envelope disintegration. The dense hydrophobic mycolate layer which normally confers resistance to hydrophilic antibiotics may become susceptible in the presence of amphipathic fatty acids. Insertion of DHA likely increases membrane fluidity, induces phase separation, and compromises barrier function (Mostofian et al., 2019).

For the isolate *N. cyriacigeorgica* CY040 used in transcriptomic analysis, all four active EFAs (ALA, GLA, EPA, and DHA) showed identical MIC values (31.25 μM). DHA was selected for mechanistic studies because its longer carbon chain and higher unsaturation are known to confer strong membrane-perturbing properties (Mason et al., 2016; Sherratt et al., 2023).

The transcriptional changes observed in DHA-treated *N. cyriacigeorgica* CY040, characterized by downregulation of the proton-pumping Type I NADH dehydrogenase (Nuo complex) and upregulation of the non-proton-pumping Type II enzyme Ndh, recapitulates a conserved transcriptional pattern among actinobacteria. In *Mycobacterium tuberculosis*, genetic dissection of the three membrane-bound NADH dehydrogenases has demonstrated that single deletions are well tolerated, whereas simultaneous loss of *nuo* and *ndh* profoundly impairs viability and virulence (Vilchèze et al., 2018). This synthetic lethality highlights that Ndh is the principal type II NADH dehydrogenase required for fitness, as the presence of NdhA alone cannot compensate for the combined loss of Nuo and Ndh. Remarkably, the transcriptional changes induced by DHA, characterized by downregulation of the Nuo complex and upregulation of Ndh, recapitulate the very state in which the bacterium becomes dependent on the type II enzyme as a compensatory respiratory node. Our findings, together with the established genetic interdependence of NDH-1 and NDH-2 in *M. tuberculosis*, support a unified model in which co-inhibiting both complexes represents a broadly effective strategy against mycolic acid-rich actinobacterial pathogens. The downregulation of *nuo* genes and upregulation of *ndh* suggest an altered respiratory gene expression profile, which may create a therapeutic window for combined inhibition of this backup enzyme and provide a rational basis for combination therapy.

In CY040, there was concurrently a global upregulation of genes involved in translation and amino acid biosynthesis. This upregulation of translation-associated genes may reflect a cellular response to membrane stress, potentially including the need to replace damaged proteins and thereby altering the cell’s vulnerability to protein synthesis inhibitors.

Several additional pathways were enriched in the transcriptomic data (**Supplementary Tables 1, 2**). For instance, genes involved in cation transport (GO:0006812, 82.3% upregulated) were significantly enriched, which may reflect a cellular response to maintain ion homeostasis. The biosynthesis of siderophore group nonribosomal peptides (KEGG: ncy01053, 100% upregulated) was uniformly increased, which is consistent with a role in iron acquisition. Other enriched terms, such as tetrapyrrole and porphyrin metabolism, play central roles in energy metabolism and oxidative stress responses.

Notably, DHA treatment also altered the expression of genes involved in mycolic acid biosynthesis. Upstream genes were consistently downregulated, including *pccB*, which encodes propionyl CoA carboxylase responsible for malonyl CoA synthesis in *Nocardia* and is functionally equivalent to *accD6* in *Mycobacterium tuberculosis* (Gygax et al., 1984; Kimura et al., 1998), as well as *accD4* and *pks13*. In contrast, downstream processing genes such as *cmrA* and *ag85C* were upregulated, suggesting an attempt by the bacterium to salvage residual lipid intermediates for envelope repair. This distinct expression pattern, involving suppression of upstream genes and activation of downstream genes, reflects a specific transcriptional response of the mycolic acid biosynthesis pathway to DHA treatment.

Collectively, these transcriptional changes indicate that surviving bacteria undergo a coordinated response. Whether this response leads to increased susceptibility to antibiotics requires experimental validation. As described in Section 2.4, *N. wallacei* was selected for combination testing due to its intrinsic high-level resistance to aminoglycosides mediated by the chromosomally encoded 16S rRNA methyltransferase WarA (Lowman and Aithma, 2010; Hershko et al., 2024). In contrast, susceptibility testing revealed that the other clinical isolates in our collection remained susceptible to amikacin (MICs ≤ 16 μg/mL) (Wei et al., 2021). Thus, *N. wallacei* represents a clinically challenging scenario in which conventional aminoglycoside therapy is ineffective, making it a critical model for evaluating whether DHA can restore drug susceptibility through membrane permeabilization.

Our observation that sub-inhibitory DHA enhances the activity of amikacin against *N. wallacei* isolates provides experimental support for this combination strategy. Membrane disruption likely facilitates increased intracellular accumulation of aminoglycosides, thereby lowering their effective concentrations. This effect was more pronounced for amikacin than for tobramycin. In *N. wallacei*, aminoglycoside resistance is primarily mediated by WarA, a 16S rRNA methyltransferase that introduces a methyl group at position m¹A1408, thereby reducing drug binding to the ribosome (Hershko et al., 2023; Hershko et al., 2024). Notably, amikacin, owing to its unique 4-amino-2-hydroxybutyryl (AHB) side chain, forms additional contacts with the ribosomal decoding center that are not available to other aminoglycosides (Seely et al., 2023). This expanded interaction profile may confer partial activity against methylated ribosomes, whereas tobramycin binding is more completely abolished by 16S rRNA methylation. The differential effects observed between the consistent potentiation of amikacin and the only occasional enhancement of tobramycin may therefore reflect differences in the intrinsic ability of each drug to engage methylated ribosomal targets. Further studies are needed to determine whether DHA also impairs WarA-mediated methylation under the conditions used here, which would provide an additional mechanistic layer underlying the observed additive effect.

Limitations include the single time point of transcriptomic analysis, which captures early but not late transcriptional responses, and the high-biomass condition that does not incorporate host immune factors or pharmacokinetic variables. Furthermore, because the transcriptomic analysis relied on a single reference genome (GUH-2), reads from accessory genes or genomic islands unique to CY040 cannot be mapped, which may lead to an underestimation of expression changes in variable genomic regions. However, the accuracy of expression changes for core genes central to our conclusions is not compromised by this limitation, as these genes are well-represented in the reference genome. Additionally, direct lipid analysis is needed to determine whether DHA affects mycolic acid content. Functional validation of the respiratory shift and *in vivo* efficacy studies in animal infection models will further support the translational potential.

In summary, DHA exerts rapid bactericidal activity against clinical *Nocardia* isolates by disrupting the cell envelope and triggering a broad transcriptional response. In the surviving subpopulation, we observed altered expression of genes involved in respiration, protein synthesis, and mycolic acid biosynthesis. This transcriptional pattern, while potentially enabling short-term survival, may create vulnerabilities that could be therapeutically exploited. This membrane-targeting approach holds promise for treating persistent infections caused by mycolic acid-containing, slow-growing bacterial pathogens.

## Data Availability

The datasets presented in this study can be found in online repositories. The names of the repository/repositories and accession number(s) can be found in the article/**Supplementary Material**.
