# Supplementary material for: Essential fatty acids disrupt the mycolic acid-rich cell envelope of clinical Nocardia isolates: a membrane-targeting approach to combat persistent infections
Source: Front Cell Infect Microbiol. 2026 Aug 7;16:1878912. doi: 10.3389/fcimb.2026.1878912 (PMC13493296; doi:10.3389/fcimb.2026.1878912)
Supplement: Supplementary file 1 [file Table1.docx]

Supplementary Material

**Table S1** The percentage of upregulated genes in GO enrichment analysis

| **Category** | **GO ID** | **Description** | **Percentage of upregulated genes (****upregulated / total DEGs in GO term)** |
| --- | --- | --- | --- |
| BP | GO:0009142 | nucleoside triphosphate biosynthetic process | 100% (8 / 8) |
| BP | GO:0046034 | ATP metabolic process | 100% (8 / 8) |
| BP | GO:0033014 | tetrapyrrole biosynthetic process | 100% (9 / 9) |
| BP | GO:0043604 | amide biosynthetic process | 100% (37 / 37) |
| BP | GO:0043603 | cellular amide metabolic process | 100% (38 / 38) |
| BP | GO:0006790 | sulfur compound metabolic process | 80.0% (8 / 10) |
| BP | GO:0033013 | tetrapyrrole metabolic process | 100% (9 / 9) |
| BP | GO:0006754 | ATP biosynthetic process | 100% (7 / 7) |
| BP | GO:0009145 | purine nucleoside triphosphate biosynthetic process | 100% (7 / 7) |
| BP | GO:0009201 | ribonucleoside triphosphate biosynthetic process | 100% (7 / 7) |
| BP | GO:0009206 | purine ribonucleoside triphosphate biosynthetic process | 100% (7 / 7) |
| BP | GO:1901566 | organonitrogen compound biosynthetic process | 90.7% (68 / 75) |
| BP | GO:0006812 | cation transport | 82.3% (14 / 17) |
| BP | GO:0006412 | translation | 100% (33 / 33) |
| BP | GO:0043043 | peptide biosynthetic process | 100% (33 / 33) |
| CC | GO:0043228 | non-membrane-bounded organelle | 100% (27 / 27) |
| CC | GO:0043232 | intracellular non-membrane-bounded organelle | 100% (27 / 27) |
| CC | GO:0005840 | ribosome | 100% (26 / 26) |
| CC | GO:0043226 | organelle | 100% (27 / 27) |
| CC | GO:0043229 | intracellular organelle | 100% (27 / 27) |
| CC | GO:1990904 | ribonucleoprotein complex | 100% (26 / 26) |
| CC | GO:0044444 | cytoplasmic part | 100% (26 / 26) |
| CC | GO:0005622 | intracellular | 100% (34 / 34) |
| CC | GO:0044424 | intracellular part | 100% (34 / 34) |
| CC | GO:0032991 | protein-containing complex | 97.3% (36 / 37) |
| CC | GO:0005737 | cytoplasm | 100% (29 / 29) |
| CC | GO:0005623 | cell | 95.3% (41 / 43) |
| CC | GO:0044464 | cell part | 95.3% (41 / 43) |
| MF | GO:0003924 | GTPase activity | 66.7% (6 / 9) |
| MF | GO:0003735 | structural constituent of ribosome | 100% (27 / 27) |
| MF | GO:0005198 | structural molecule activity | 100% (27 / 27) |
| MF | GO:0005506 | iron ion binding | 42.1% (8 / 19) |
| MF | GO:0001882 | nucleoside binding | 75.0% (9 / 12) |
| MF | GO:0001883 | purine nucleoside binding | 75.0% (9 / 12) |
| MF | GO:0005525 | GTP binding | 75.0% (9 / 12) |
| MF | GO:0019001 | guanyl nucleotide binding | 75.0% (9 / 12) |
| MF | GO:0032549 | ribonucleoside binding | 75.0% (9 / 12) |
| MF | GO:0032550 | purine ribonucleoside binding | 75.0% (9 / 12) |
| MF | GO:0032561 | guanyl ribonucleotide binding | 75.0% (9 / 12) |
| MF | GO:0016829 | lyase activity | 63.6% (14 / 22) |
| MF | GO:0046914 | transition metal ion binding | 51.6% (16 / 31) |
| MF | GO:0016741 | transferase activity, transferring one-carbon groups | 73.9% (17 / 23) |

**Table S2** The percentage of upregulated genes in KEGG enrichment analysis

| **KEGG ID** | **Description** | **Percentage of upregulated genes (upregulated / total DEGs in KEGG pathway)** |
| --- | --- | --- |
| ncy03010 | Ribosome | 100% (34 / 34) |
| ncy01120 | Microbial metabolism in diverse environments | 59.2% (61 / 103) |
| ncy00770 | Pantothenate and CoA biosynthesis | 76.9% (10 / 13) |
| ncy00860 | Porphyrin metabolism | 90.5% (19 / 21) |
| ncy00071 | Fatty acid degradation | 54.2% (13 / 24) |
| ncy00190 | Oxidative phosphorylation | 72.0% (18 / 25) |
| ncy01110 | Biosynthesis of secondary metabolites | 76.8% (119 / 155) |
| ncy01053 | Biosynthesis of siderophore group nonribosomal peptides | 100% (8 / 8) |

**Table S3** Differentially expressed respiratory genes in DHA‑treated *N. cyriacigeorgica*

| **Gene ID** | **Gene name** | **log_2_FoldChange** | **Gene description (Genome && SwissProt && Pfam)** |
| --- | --- | --- | --- |
| NOCYR_RS16170 | *nuoA* | -2.98 | NADH-quinone oxidoreductase subunit A && sp\|P65564\|NUOA_MYCBO NADH-quinone oxidoreductase subunit A OS=Mycobacterium bovis (strain ATCC BAA-935 / AF2122/97) OX=233413 GN=nuoA PE=3 SV=1 && PF00507:NADH-ubiquinone/plastoquinone oxidoreductase, chain 3 |
| NOCYR_RS16175 | *nuoB* | -2.75 | NuoB/complex I 20 kDa subunit family protein && sp\|Q5YWC8\|NUOB_NOCFA NADH-quinone oxidoreductase subunit B OS=Nocardia farcinica (strain IFM 10152) OX=247156 GN=nuoB PE=3 SV=1 && PF01058:NADH ubiquinone oxidoreductase, 20 Kd subunit |
| NOCYR_RS16180 | *nuoC* | -2.55 | NADH-quinone oxidoreductase subunit C && sp\|Q5YWC9\|NUOC_NOCFA NADH-quinone oxidoreductase subunit C OS=Nocardia farcinica (strain IFM 10152) OX=247156 GN=nuoC PE=3 SV=1 && PF00329:Respiratory-chain NADH dehydrogenase, 30 Kd subunit |
| NOCYR_RS16185 | *nuoD* | -2.57 | NADH dehydrogenase (quinone) subunit D && sp\|Q5YWD0\|NUOD_NOCFA NADH-quinone oxidoreductase subunit D OS=Nocardia farcinica (strain IFM 10152) OX=247156 GN=nuoD PE=3 SV=1 && PF00346:Respiratory-chain NADH dehydrogenase, 49 Kd subunit |
| NOCYR_RS16190 | *nuoE* | -1.97 | NADH-quinone oxidoreductase subunit NuoE && sp\|P65574\|NUOE_MYCBO NADH-quinone oxidoreductase subunit E OS=Mycobacterium bovis (strain ATCC BAA-935 / AF2122/97) OX=233413 GN=nuoE PE=3 SV=1 && PF01257:Thioredoxin-like [2Fe-2S] ferredoxin |
| NOCYR_RS16195 | *nuoF* | -1.33 | NADH-quinone oxidoreductase subunit NuoF && sp\|P65568\|NUOF_MYCBO NADH-quinone oxidoreductase subunit F OS=Mycobacterium bovis (strain ATCC BAA-935 / AF2122/97) OX=233413 GN=nuoF PE=3 SV=1 && PF01512:Nuo51 FMN-binding domain\|PF10589:NADH-ubiquinone oxidoreductase-F iron-sulfur binding region |
| NOCYR_RS20190 | *ndh* | 4.51 | NAD(P)/FAD-dependent oxidoreductase && sp\|P95160\|NDH_MYCTU Type II NADH:quinone oxidoreductase Ndh OS=Mycobacterium tuberculosis (strain ATCC 25618 / H37Rv) OX=83332 GN=ndh PE=1 SV=1 && PF07992:Pyridine nucleotide-disulphide oxidoreductase |

**Table S4** Differentially expressed mycolic acid biosynthesis genes in DHA‑treated *N. cyriacigeorgica*

| **Gene ID** | **Gene name** | **log_2_FoldChange** | **Gene description** **(Genome && SwissProt && Pfam)** |
| --- | --- | --- | --- |
| NOCYR_RS08400 | *pccB* | -1.27 | acyl-CoA carboxylase subunit beta && sp\|Q06101\|PCCB_RHOER Propionyl-CoA carboxylase beta chain OS=Rhodococcus erythropolis OX=1833 GN=pccB PE=3 SV=1 && PF01039:Carboxyl transferase domain |
| NOCYR_RS00765 | *accD4* | -1.71 | acyl-CoA carboxylase subunit beta && sp\|O53578\|ACCD4_MYCTU Biotin-dependent long chain acyl-coenzyme A carboxylase beta4 subunit OS=Mycobacterium tuberculosis (strain ATCC 25618 / H37Rv) OX=83332 GN=accD4 PE=1 SV=2 && PF01039:Carboxyl transferase domain |
| NOCYR_RS08390 | *acpM* | -1.15 | meromycolate extension acyl carrier protein AcpM && sp\|A0R0B3\|ACPM_MYCS2 Meromycolate extension acyl carrier protein OS=Mycolicibacterium smegmatis (strain ATCC 700084 / mc(2)155) OX=246196 GN=acpM PE=1 SV=1 && PF00550:Phosphopantetheine attachment site |
| NOCYR_RS08385 | *fabD* | -1.19 | ACP S-malonyltransferase && sp\|P63459\|FABD_MYCBO Malonyl CoA-acyl carrier protein transacylase OS=Mycobacterium bovis (strain ATCC BAA-935 / AF2122/97) OX=233413 GN=fabD PE=3 SV=1 && PF00698:Acyl transferase domain |
| NOCYR_RS26900 | *fabH* | -4.13 | 3-oxoacyl-ACP synthase III family protein && sp\|P59814\|FABH_RHOBA Beta-ketoacyl-[acyl-carrier-protein] synthase III OS=Rhodopirellula baltica (strain DSM 10527 / NCIMB 13988 / SH1) OX=243090 GN=fabH PE=3 SV=1 && PF08541:3-Oxoacyl-[acyl-carrier-protein (ACP)] synthase III C terminal |
| NOCYR_RS08200 | *fabH1* | 2.30 | beta-ketoacyl-ACP synthase III && sp\|Q82BB8\|FABH1_STRAW Beta-ketoacyl-[acyl-carrier-protein] synthase III 1 OS=Streptomyces avermitilis (strain ATCC 31267 / DSM 46492 / JCM 5070 / NBRC 14893 / NCIMB 12804 / NRRL 8165 / MA-4680) OX=227882 GN=fabH1 PE=3 SV=1 && PF08545:3-Oxoacyl-[acyl-carrier-protein (ACP)] synthase III\|PF08541:3-Oxoacyl-[acyl-carrier-protein (ACP)] synthase III C terminal |
| NOCYR_RS13410 | *hadA/hadB* | -3.27 | fused (3R)-hydroxyacyl-ACP dehydratase subunits HadA/HadB && sp\|Q9CBJ6\|Y1910_MYCLE UPF0336 protein ML1910 OS=Mycobacterium leprae (strain TN) OX=272631 GN=ML1910 PE=3 SV=1 && PF01575:MaoC like domain\|PF13452:FAS1-like, dehydratase domain region |
| NOCYR_RS13440 | *hadA/hadB* | -3.07 | fused (3R)-hydroxyacyl-ACP dehydratase subunits HadA/HadB && sp\|Q73SS7\|Y3996_MYCPA UPF0336 protein MAP_3996c OS=Mycolicibacterium paratuberculosis (strain ATCC BAA-968 / K-10) OX=262316 GN=MAP_3996c PE=3 SV=1 && PF13452:FAS1-like, dehydratase domain region\|PF01575:MaoC like domain |
| NOCYR_RS00760 | *pks13* | -1.19 | polyketide synthase Pks13 && sp\|I6X8D2\|PKS13_MYCTU Polyketide synthase Pks13 OS=Mycobacterium tuberculosis (strain ATCC 25618 / H37Rv) OX=83332 GN=pks13 PE=1 SV=1 && PF00550:Phosphopantetheine attachment site\|PF02801:Beta-ketoacyl synthase, C-terminal domain\|PF00698:Acyl transferase domain\|PF00975:Thioesterase domain\|PF00109:Beta-ketoacyl synthase, N-terminal domain\|PF16197:Ketoacyl-synthetase C-terminal extension |
| NOCYR_RS06485 | *cmrA* | 1.24 | mycolate reductase && sp\|P0AFP5\|YBBO_ECOL6 Uncharacterized oxidoreductase YbbO OS=Escherichia coli O6:H1 (strain CFT073 / ATCC 700928 / UPEC) OX=199310 GN=ybbO PE=3 SV=1 && PF00106:short chain dehydrogenase |
| NOCYR_RS00730 | *ag85C* | 2.26 | alpha/beta hydrolase && sp\|P0A4V5\|A85C_MYCBO Diacylglycerol acyltransferase/mycolyltransferase Ag85C OS=Mycobacterium bovis (strain ATCC BAA-935 / AF2122/97) OX=233413 GN=fbpC PE=3 SV=1 && PF00756:Putative esterase |
| NOCYR_RS11300 | *ag85C* | 2.52 | alpha/beta hydrolase && sp\|O52972\|A85C_MYCAV Diacylglycerol acyltransferase/mycolyltransferase Ag85C OS=Mycobacterium avium OX=1764 GN=fbpC PE=3 SV=1 && PF00756:Putative esterase |
| NOCYR_RS09630 | *-* | -1.88 | class I SAM-dependent methyltransferase && - && PF08241:Methyltransferase domain |
| NOCYR_RS02870 | *-* | -4.27 | class I SAM-dependent methyltransferase && sp\|O53532\|Y2258_MYCTU S-adenosylmethionine-dependent methyltransferase Rv2258c OS=Mycobacterium tuberculosis (strain ATCC 25618 / H37Rv) OX=83332 GN=Rv2258c PE=1 SV=1 && PF21320:Rv2258c-like winged HTH domain\|PF13847:Methyltransferase domain |
| NOCYR_RS15795 | *-* | -1.95 | SAM-dependent methyltransferase && - && PF04672:S-adenosyl methyltransferase |
| NOCYR_RS20260 | *-* | 1.14 | class I SAM-dependent methyltransferase && sp\|A9ILA7\|UBIE_BART1 Ubiquinone/menaquinone biosynthesis C-methyltransferase UbiE OS=Bartonella tribocorum (strain CIP 105476 / IBS 506) OX=382640 GN=ubiE PE=3 SV=1 && PF08241:Methyltransferase domain |
| NOCYR_RS20225 | *-* | 1.47 | class I SAM-dependent methyltransferase && sp\|A0QUV5\|Y2350_MYCS2 Probable S-adenosylmethionine-dependent methyltransferase MSMEG_2350/MSMEI_2290 OS=Mycolicibacterium smegmatis (strain ATCC 700084 / mc(2)155) OX=246196 GN=MSMEG_2350 PE=1 SV=1 && PF08241:Methyltransferase domain |
| NOCYR_RS16660 | *-* | -1.31 | class I SAM-dependent methyltransferase && - && - |
| NOCYR_RS17515 | *-* | -1.41 | SAM-dependent methyltransferase && sp\|A8LHA4\|Y4929_PARS2 Putative S-adenosyl-L-methionine-dependent methyltransferase Franean1_4929 OS=Parafrankia sp. (strain EAN1pec) OX=298653 GN=Franean1_4929 PE=3 SV=2 && PF04072:Leucine carboxyl methyltransferase |
| NOCYR_RS16750 | *-* | 1.48 | class I SAM-dependent methyltransferase && - && - |
| NOCYR_RS25030 | *-* | 3.18 | class I SAM-dependent methyltransferase && - && - |
